# Supplementary material for: The comprehensive analysis of the prognostic and functional role of N-terminal methyltransferases 1 in pan-cancer
Source: PeerJ. 2023 Oct 24;11:e16263. doi: 10.7717/peerj.16263 (PMC10607204; doi:10.7717/peerj.16263)
Supplement: Supplemental Information 1 — (A) NTMT1 protein levels in variety of tumors from UALCAN. (B) Protein expression of NTMT1 across pan cancer subtype. (C) Protein expression of NTMT1 across pan cancer subtype2. [file peerj-11-16263-s001.pdf]

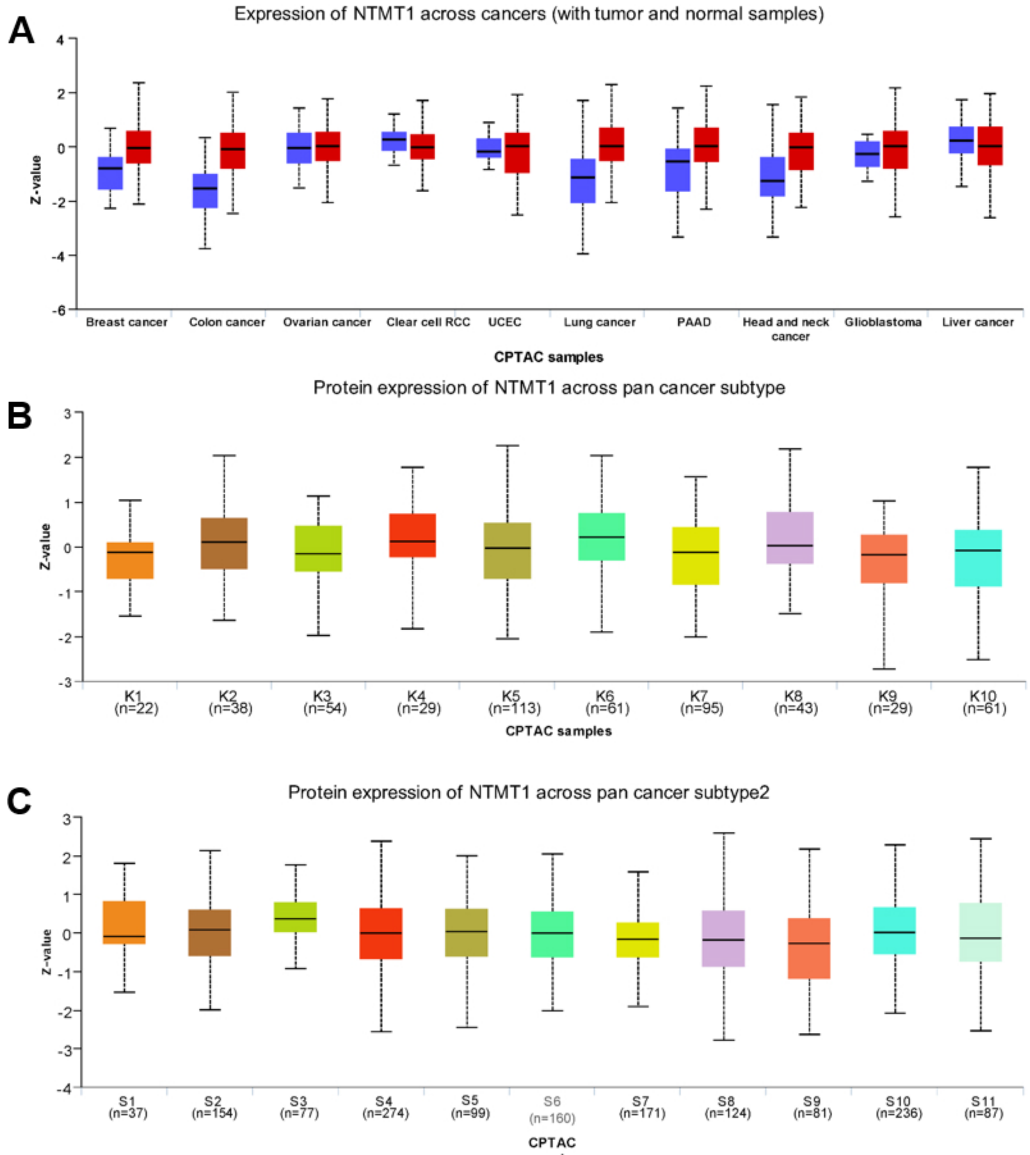
A
Expression of NTMT1 across cancers (with tumor and normal samples)
Z-value
4
2
0
-2
-4
-6
Breast cancer
Colon cancer
Ovarian cancer
Clear cell RCC
UCEC
Lung cancer
PAAD
Head and neck cancer
Glioblastoma
Liver cancer
CPTAC samples
B
Protein expression of NTMT1 across pan cancer subtype
Z-value
3
2
1
0
-1
-2
-3
K1 (n=22)
K2 (n=38)
K3 (n=54)
K4 (n=29)
K5 (n=113)
K6 (n=61)
K7 (n=95)
K8 (n=43)
K9 (n=29)
K10 (n=61)
CPTAC samples
C
Protein expression of NTMT1 across pan cancer subtype2
Z-value
3
2
1
0
-1
-2
-3
-4
S1 (n=37)
S2 (n=154)
S3 (n=77)
S4 (n=274)
S5 (n=99)
S6 (n=160)
S7 (n=171)
S8 (n=124)
S9 (n=81)
S10 (n=236)
S11 (n=87)
CPTAC
