# Supplementary figures and images for: The comprehensive analysis of the prognostic and functional role of N-terminal methyltransferases 1 in pan-cancer
Source: PeerJ. 2023 Oct 24;11:e16263. doi: 10.7717/peerj.16263 (PMC10607204; doi:10.7717/peerj.16263)

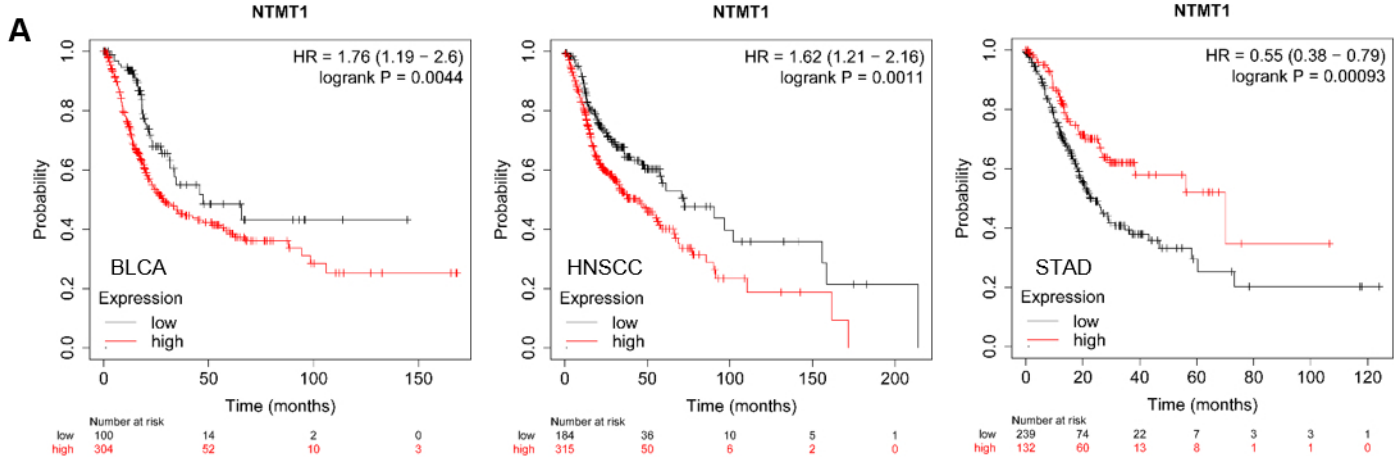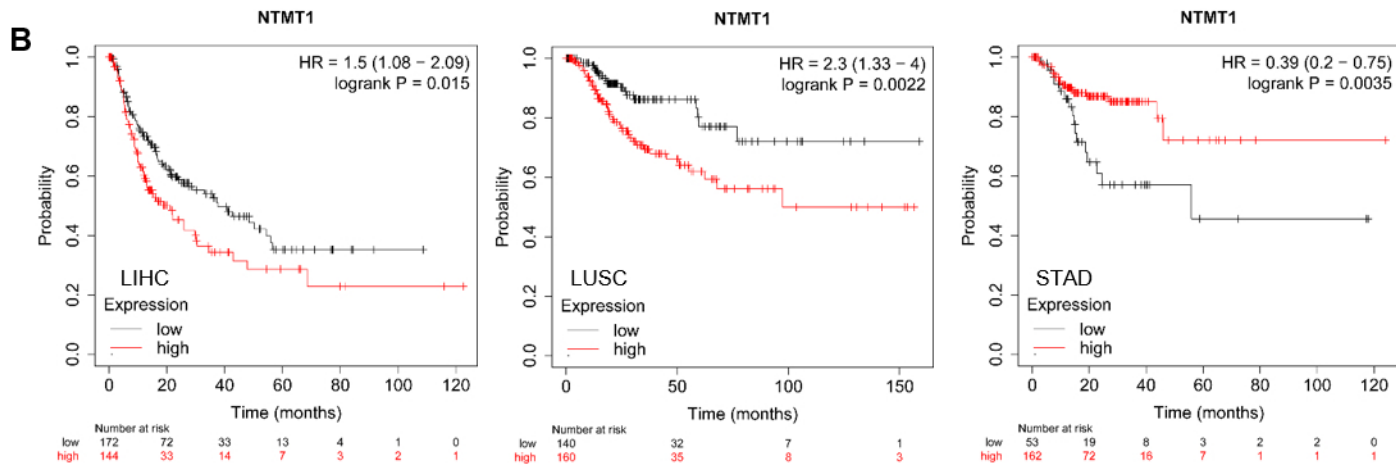

Supplement: Supplemental Information 2 — (A) Effects of NTMT1 expression on OS in BLCA, HNSCC and STAD, respectively. (B) Effects of NTMT1 expression on RFS in LIHC, LUSC and STAD, respectively. [file peerj-11-16263-s002.pdf]

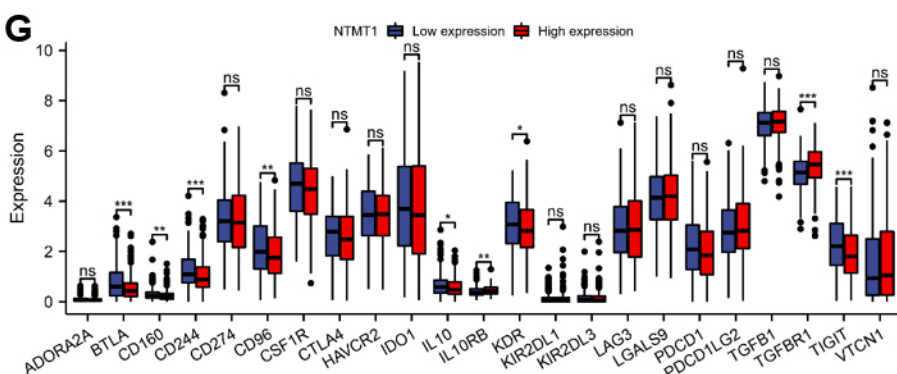

Supplement: Supplemental Information 4 — (A) The relationship of NTMT1 expression and infiltration of immune cells in HNSCC. (B) The relationship of NTMT1 expression and infiltration of stromal score in HNSCC. (C–G) The relationship of NTMT1 expression and chemokines (C), immunostimulators (D), chemokines receptors (E), MHC molecules (F), and immunoinhibitors (G) in HNSCC. ns, p＞0.05, *p＜0.05, **p＜0.01, ***p＜0.001. [file peerj-11-16263-s004.pdf]

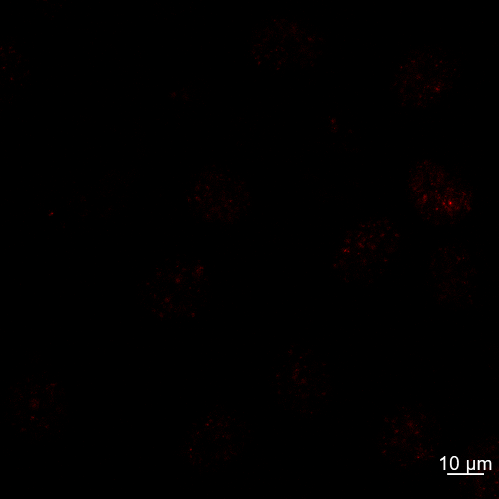

Supplement: Supplemental Information 6 — - [file peerj-11-16263-s006.zip › data/Figure 10C/siNC/siNC-10-1 represent image.tif]

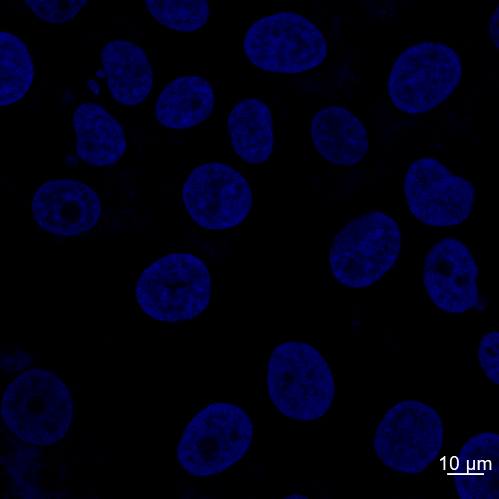

Supplement: Supplemental Information 6 — - [file peerj-11-16263-s006.zip › data/Figure 10C/siNC/siNC-10-2.tif]

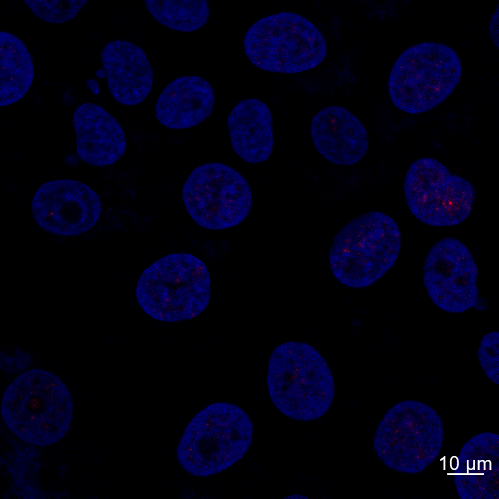

Supplement: Supplemental Information 6 — - [file peerj-11-16263-s006.zip › data/Figure 10C/siNC/siNC-10-3.tif]

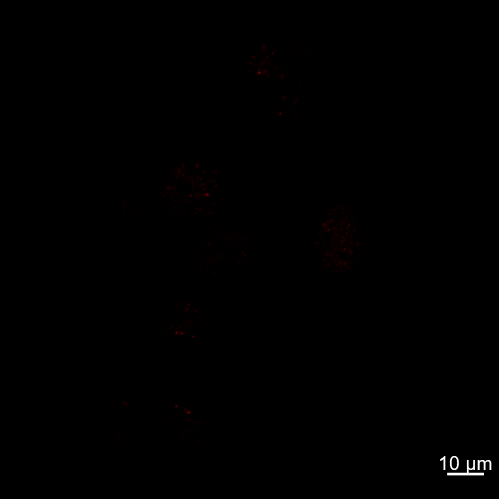

Supplement: Supplemental Information 6 — - [file peerj-11-16263-s006.zip › data/Figure 10C/siNC/siNC-6-1.tif]

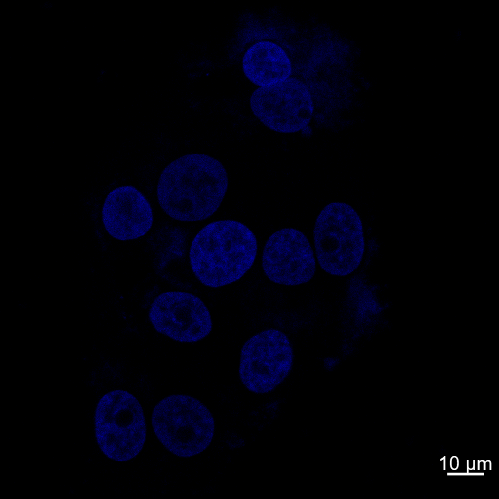

Supplement: Supplemental Information 6 — - [file peerj-11-16263-s006.zip › data/Figure 10C/siNC/siNC-6-2.tif]

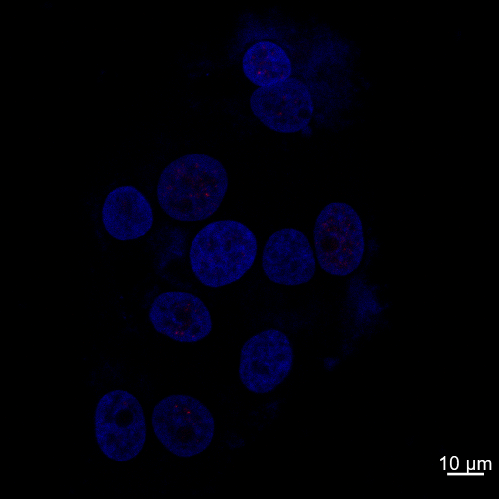

Supplement: Supplemental Information 6 — - [file peerj-11-16263-s006.zip › data/Figure 10C/siNC/siNC-6-3.tif]

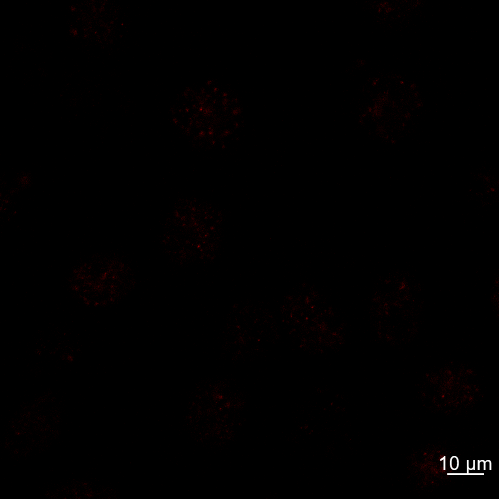

Supplement: Supplemental Information 6 — - [file peerj-11-16263-s006.zip › data/Figure 10C/siNC/siNC-7-1.tif]

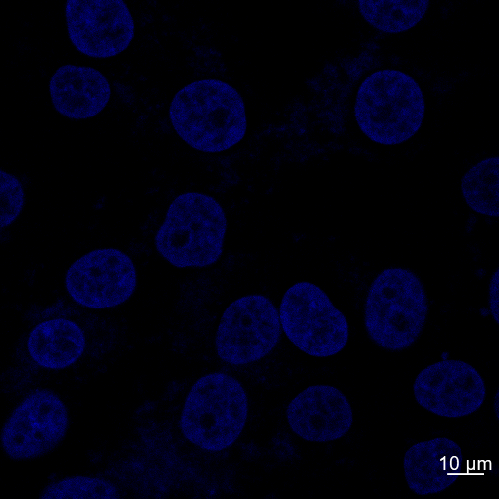

Supplement: Supplemental Information 6 — - [file peerj-11-16263-s006.zip › data/Figure 10C/siNC/siNC-7-2.tif]

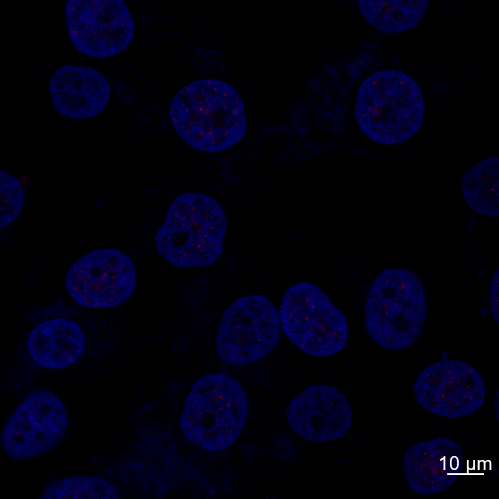

Supplement: Supplemental Information 6 — - [file peerj-11-16263-s006.zip › data/Figure 10C/siNC/siNC-7-3.tif]

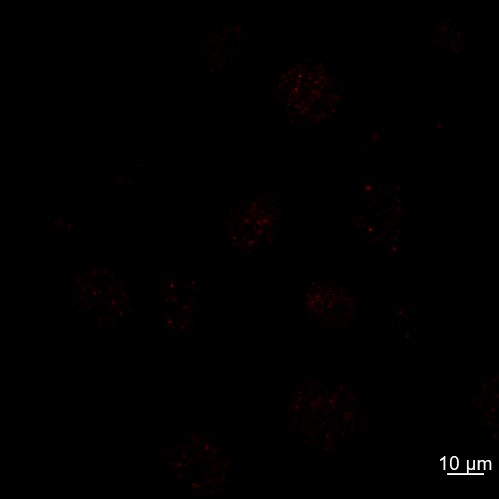

Supplement: Supplemental Information 6 — - [file peerj-11-16263-s006.zip › data/Figure 10C/siNC/siNC-8-1.tif]

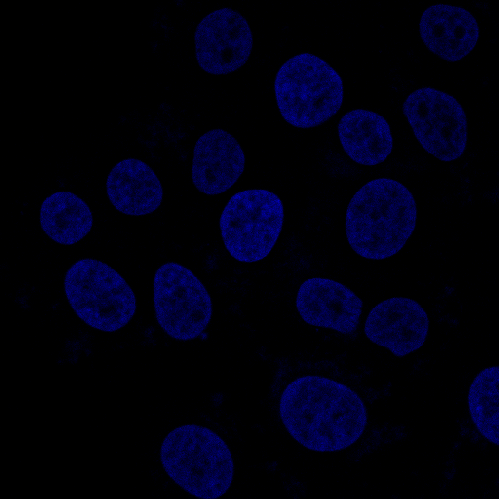

Supplement: Supplemental Information 6 — - [file peerj-11-16263-s006.zip › data/Figure 10C/siNC/siNC-8-2.tif]

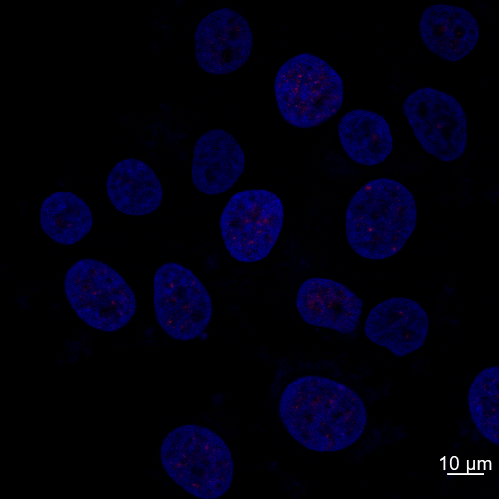

Supplement: Supplemental Information 6 — - [file peerj-11-16263-s006.zip › data/Figure 10C/siNC/siNC-8-3.tif]

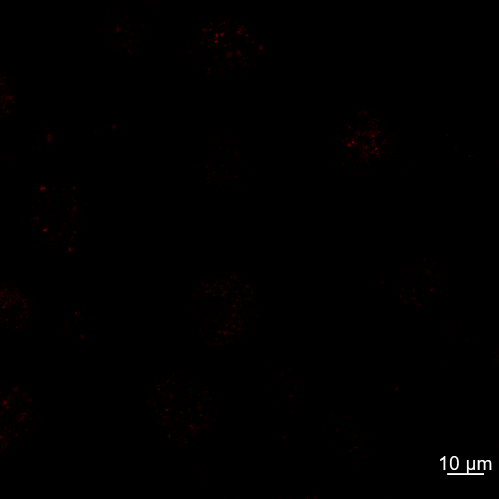

Supplement: Supplemental Information 6 — - [file peerj-11-16263-s006.zip › data/Figure 10C/siNC/siNC-9-1.tif]

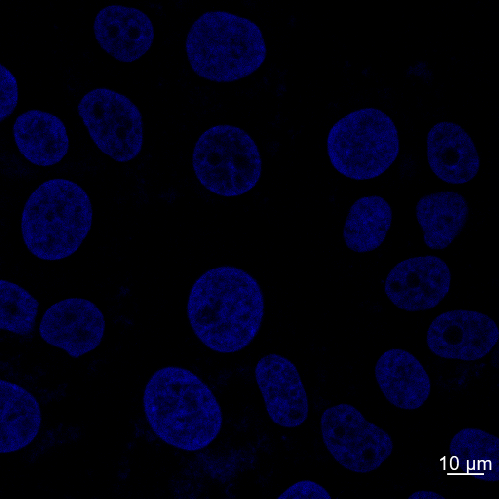

Supplement: Supplemental Information 6 — - [file peerj-11-16263-s006.zip › data/Figure 10C/siNC/siNC-9-2.tif]

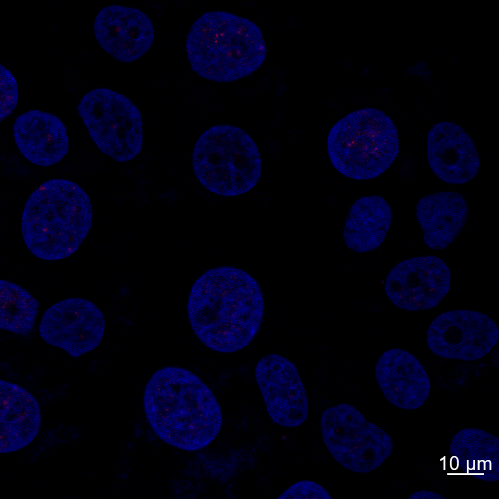

Supplement: Supplemental Information 6 — - [file peerj-11-16263-s006.zip › data/Figure 10C/siNC/siNC-9-3.tif]

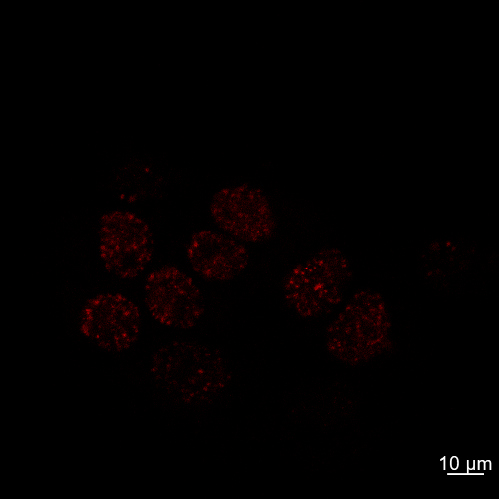

Supplement: Supplemental Information 6 — - [file peerj-11-16263-s006.zip › data/Figure 10C/siNTMT1/siNTMT1-1-1.tif]

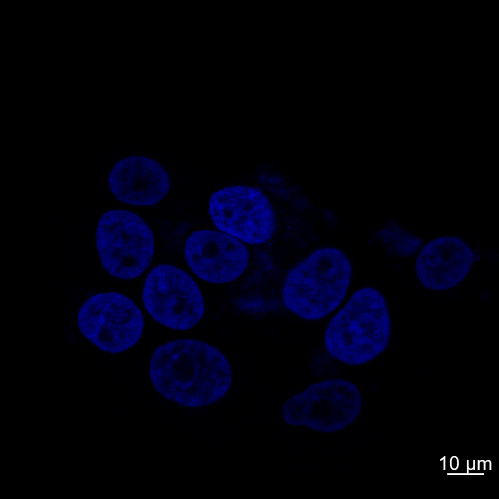

Supplement: Supplemental Information 6 — - [file peerj-11-16263-s006.zip › data/Figure 10C/siNTMT1/siNTMT1-1-2.tif]

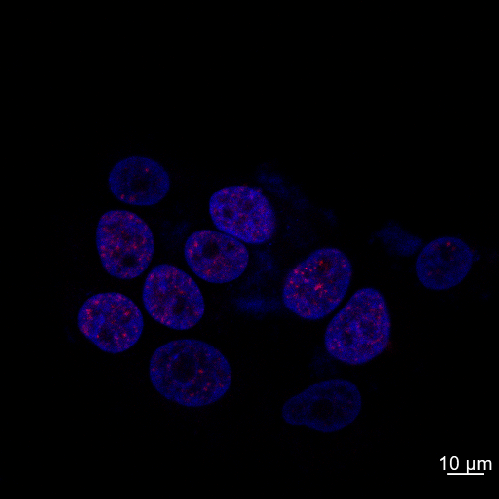

Supplement: Supplemental Information 6 — - [file peerj-11-16263-s006.zip › data/Figure 10C/siNTMT1/siNTMT1-1-3.tif]

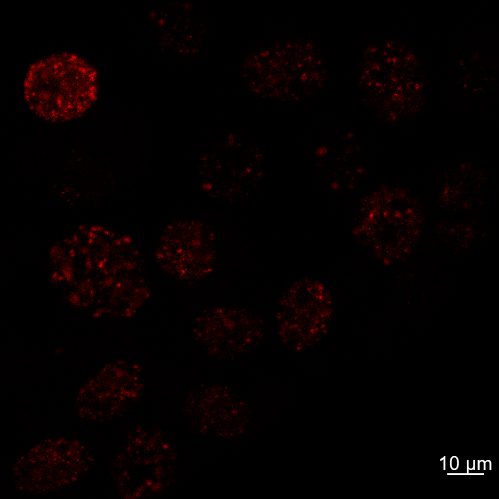

Supplement: Supplemental Information 6 — - [file peerj-11-16263-s006.zip › data/Figure 10C/siNTMT1/siNTMT1-2-1.tif]

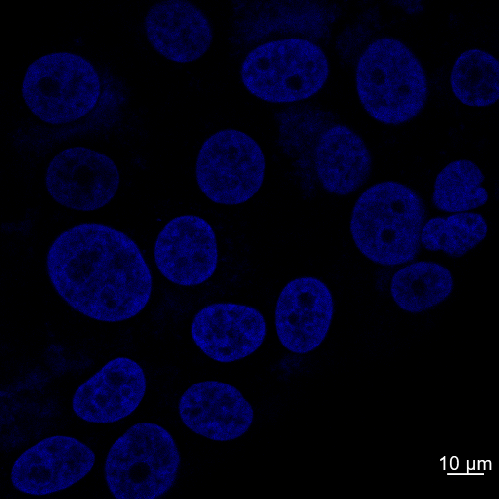

Supplement: Supplemental Information 6 — - [file peerj-11-16263-s006.zip › data/Figure 10C/siNTMT1/siNTMT1-2-2.tif]

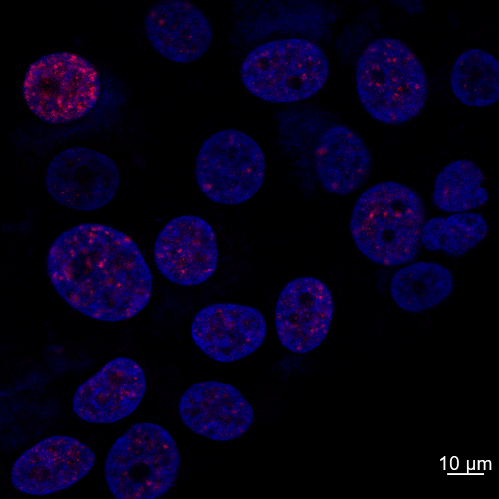

Supplement: Supplemental Information 6 — - [file peerj-11-16263-s006.zip › data/Figure 10C/siNTMT1/siNTMT1-2-3.tif]

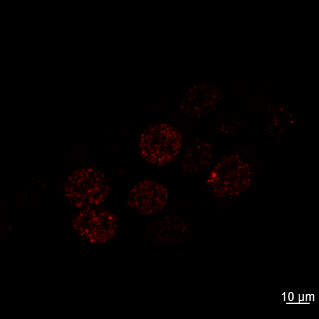

Supplement: Supplemental Information 6 — - [file peerj-11-16263-s006.zip › data/Figure 10C/siNTMT1/siNTMT1-3-1 represent image.tif]

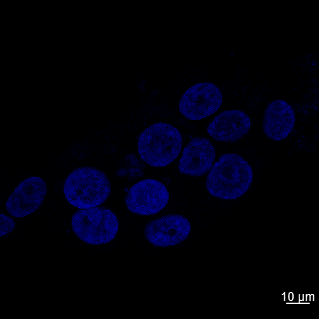

Supplement: Supplemental Information 6 — - [file peerj-11-16263-s006.zip › data/Figure 10C/siNTMT1/siNTMT1-3-2.tif]

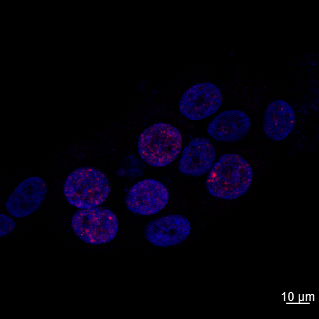

Supplement: Supplemental Information 6 — - [file peerj-11-16263-s006.zip › data/Figure 10C/siNTMT1/siNTMT1-3-3.tif]

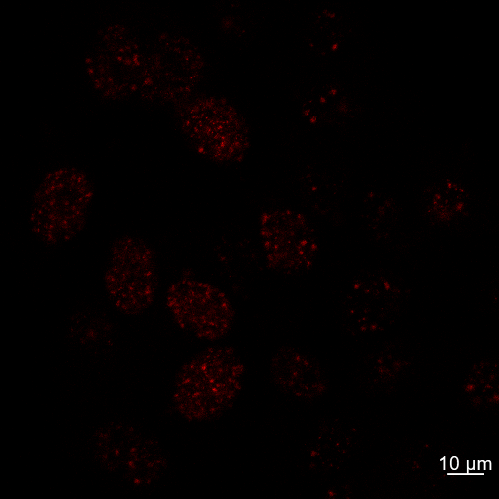

Supplement: Supplemental Information 6 — - [file peerj-11-16263-s006.zip › data/Figure 10C/siNTMT1/siNTMT1-4-1.tif]

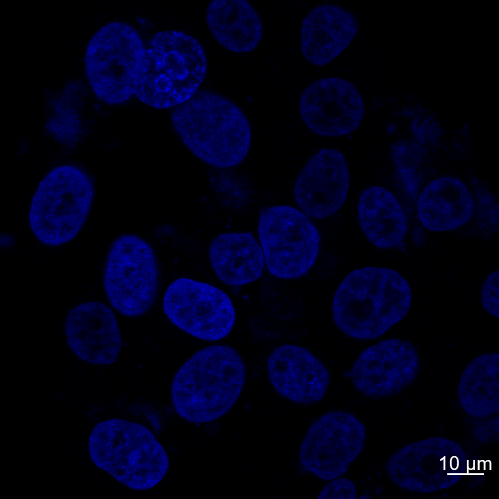

Supplement: Supplemental Information 6 — - [file peerj-11-16263-s006.zip › data/Figure 10C/siNTMT1/siNTMT1-4-2.tif]

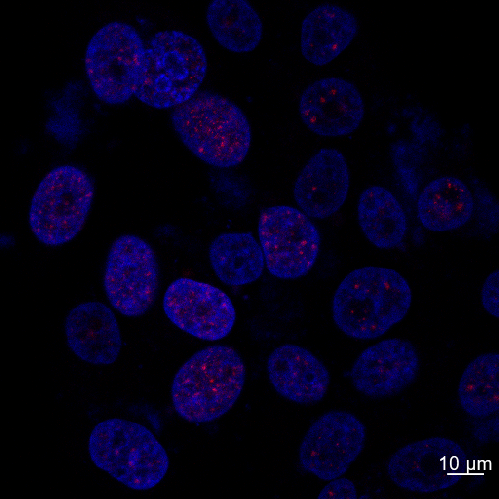

Supplement: Supplemental Information 6 — - [file peerj-11-16263-s006.zip › data/Figure 10C/siNTMT1/siNTMT1-4-3.tif]

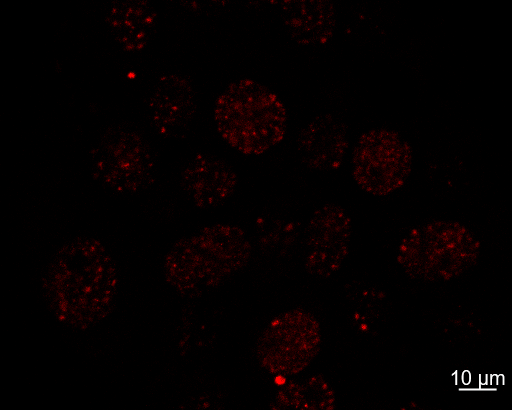

Supplement: Supplemental Information 6 — - [file peerj-11-16263-s006.zip › data/Figure 10C/siNTMT1/siNTMT1-5-1.tif]

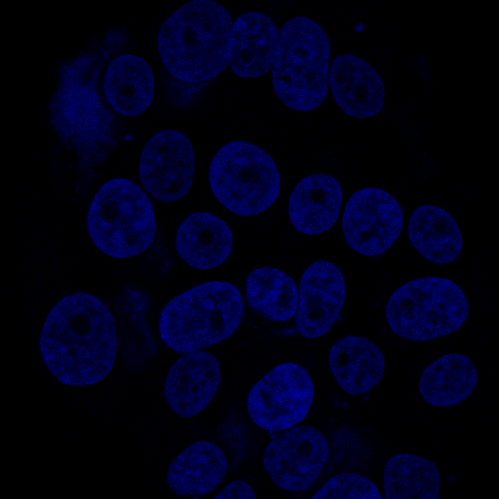

Supplement: Supplemental Information 6 — - [file peerj-11-16263-s006.zip › data/Figure 10C/siNTMT1/siNTMT1-5-2.tif]

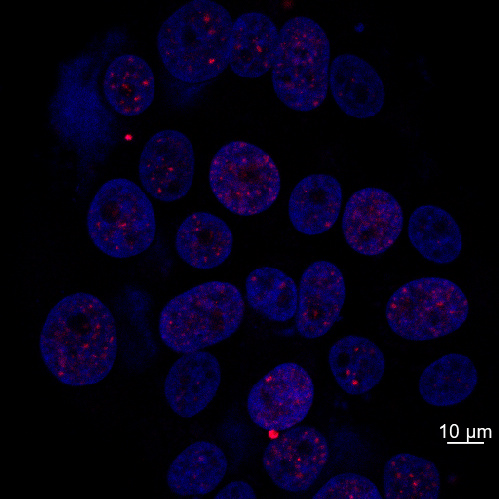

Supplement: Supplemental Information 6 — - [file peerj-11-16263-s006.zip › data/Figure 10C/siNTMT1/siNTMT1-5-3.tif]

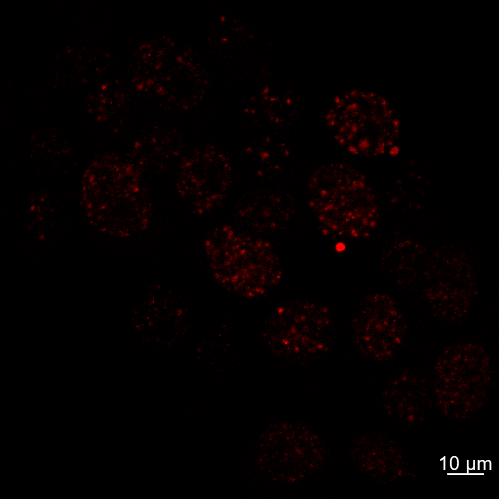

Supplement: Supplemental Information 6 — - [file peerj-11-16263-s006.zip › data/Figure 10C/siNTMT1/siNTMT1-6-1.tif]

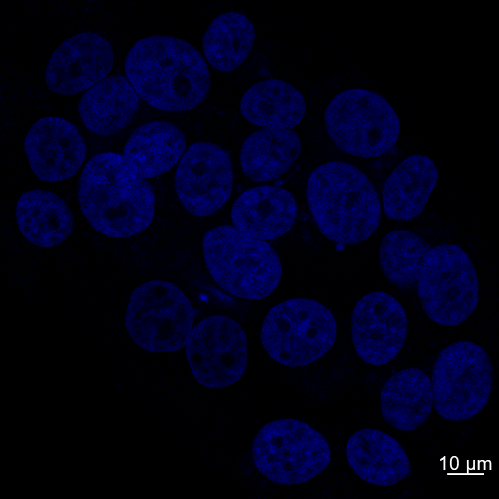

Supplement: Supplemental Information 6 — - [file peerj-11-16263-s006.zip › data/Figure 10C/siNTMT1/siNTMT1-6-2.tif]

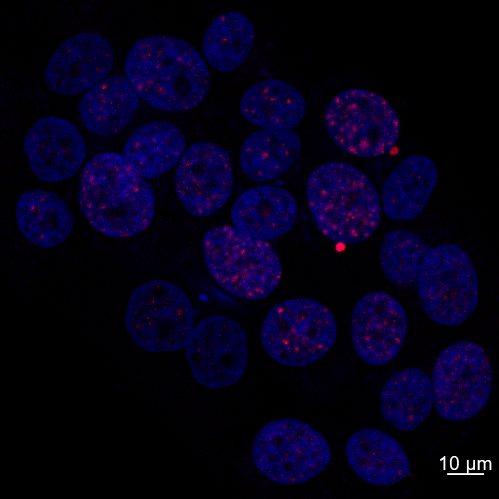

Supplement: Supplemental Information 6 — - [file peerj-11-16263-s006.zip › data/Figure 10C/siNTMT1/siNTMT1-6-3.tif]

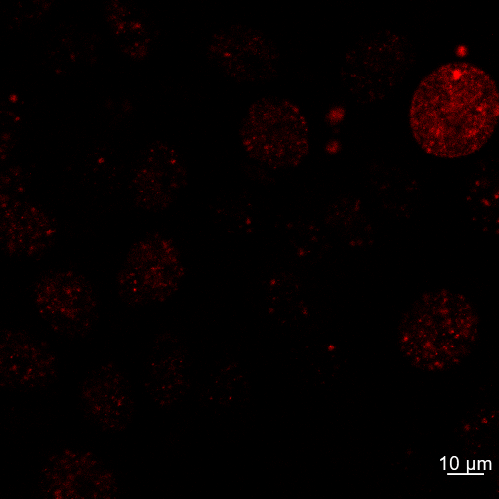

Supplement: Supplemental Information 6 — - [file peerj-11-16263-s006.zip › data/Figure 10C/siNTMT1/siNTMT1-8-1.tif]

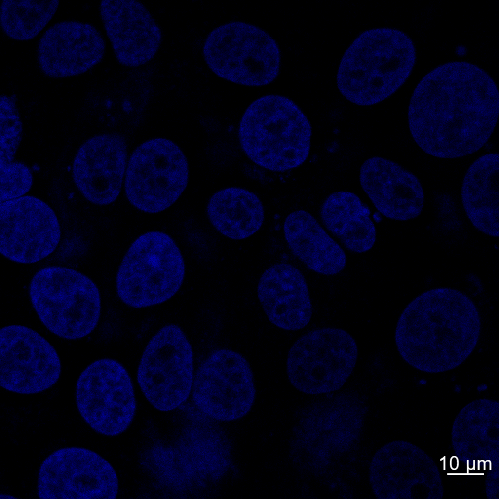

Supplement: Supplemental Information 6 — - [file peerj-11-16263-s006.zip › data/Figure 10C/siNTMT1/siNTMT1-8-2.tif]

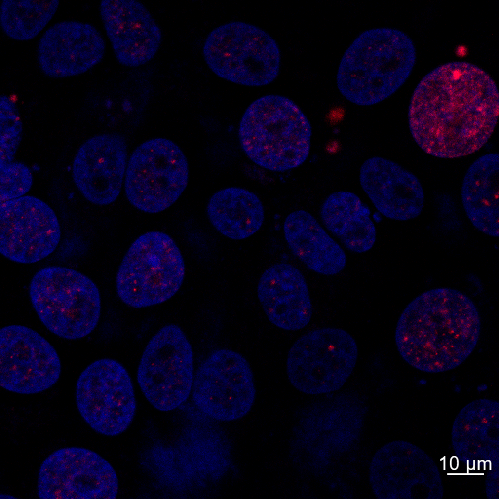

Supplement: Supplemental Information 6 — - [file peerj-11-16263-s006.zip › data/Figure 10C/siNTMT1/siNTMT1-8-3.tif]

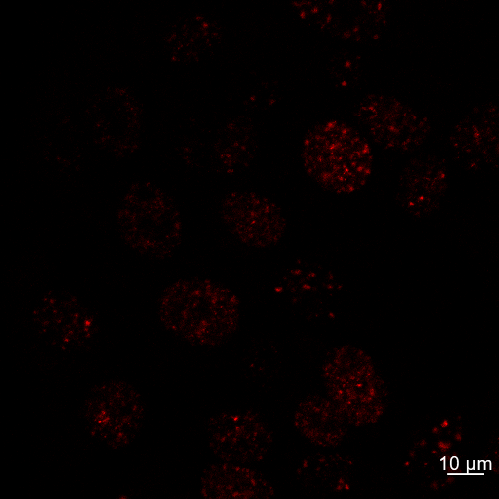

Supplement: Supplemental Information 6 — - [file peerj-11-16263-s006.zip › data/Figure 10C/siNTMT1/siNTMT1-9-1.tif]

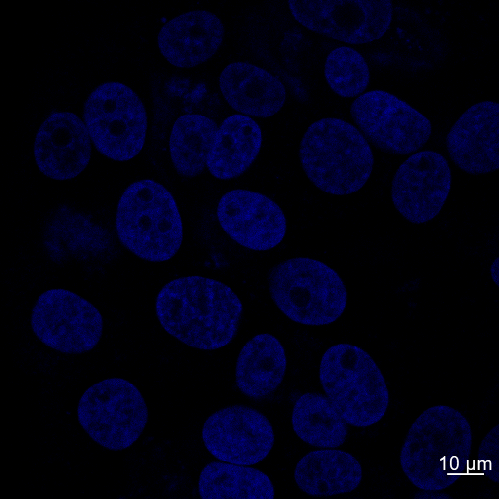

Supplement: Supplemental Information 6 — - [file peerj-11-16263-s006.zip › data/Figure 10C/siNTMT1/siNTMT1-9-2.tif]

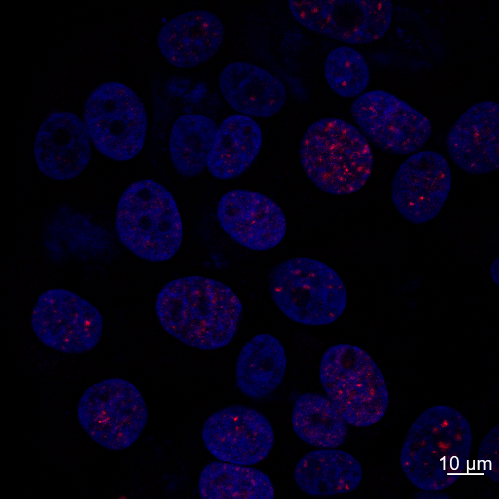

Supplement: Supplemental Information 6 — - [file peerj-11-16263-s006.zip › data/Figure 10C/siNTMT1/siNTMT1-9-3.tif]

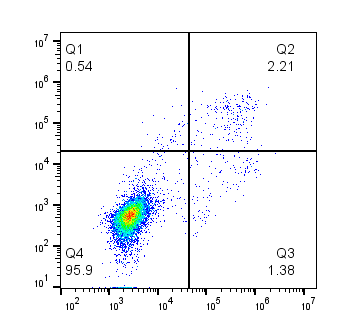

Supplement: Supplemental Information 6 — - [file peerj-11-16263-s006.zip › data/Figure 10D/siNC-1.tiff]

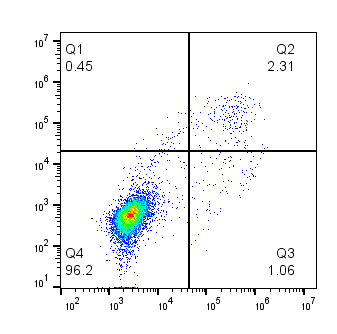

Supplement: Supplemental Information 6 — - [file peerj-11-16263-s006.zip › data/Figure 10D/siNC-2.tiff]

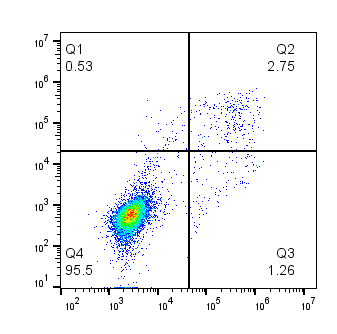

Supplement: Supplemental Information 6 — - [file peerj-11-16263-s006.zip › data/Figure 10D/siNC-3.tiff]

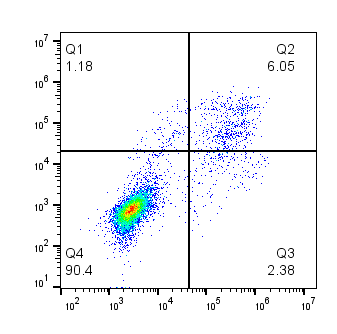

Supplement: Supplemental Information 6 — - [file peerj-11-16263-s006.zip › data/Figure 10D/siNTMT1-1.tiff]

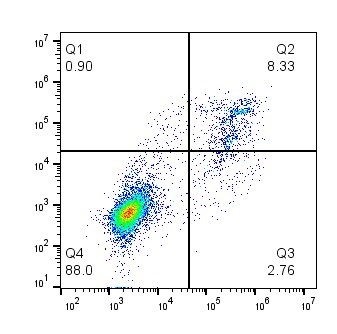

Supplement: Supplemental Information 6 — - [file peerj-11-16263-s006.zip › data/Figure 10D/siNTMT1-2.tiff]

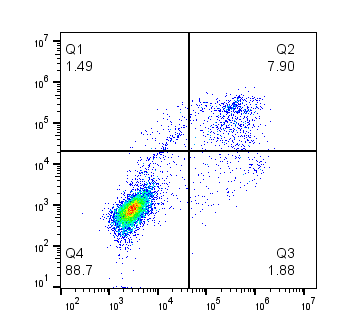

Supplement: Supplemental Information 6 — - [file peerj-11-16263-s006.zip › data/Figure 10D/siNTMT1-3.tiff]
