# Supplementary material for: The comprehensive analysis of the prognostic and functional role of N-terminal methyltransferases 1 in pan-cancer
Source: PeerJ. 2023 Oct 24;11:e16263. doi: 10.7717/peerj.16263 (PMC10607204; doi:10.7717/peerj.16263)
Supplement: Supplemental Information 3 — The methylation level of the NTMT1 promoter region in BRCA, CHOL, PAAD, LUAD, KIRP, COAD, READ, SARC, and THCA from UALCAN. [file peerj-11-16263-s003.pdf]

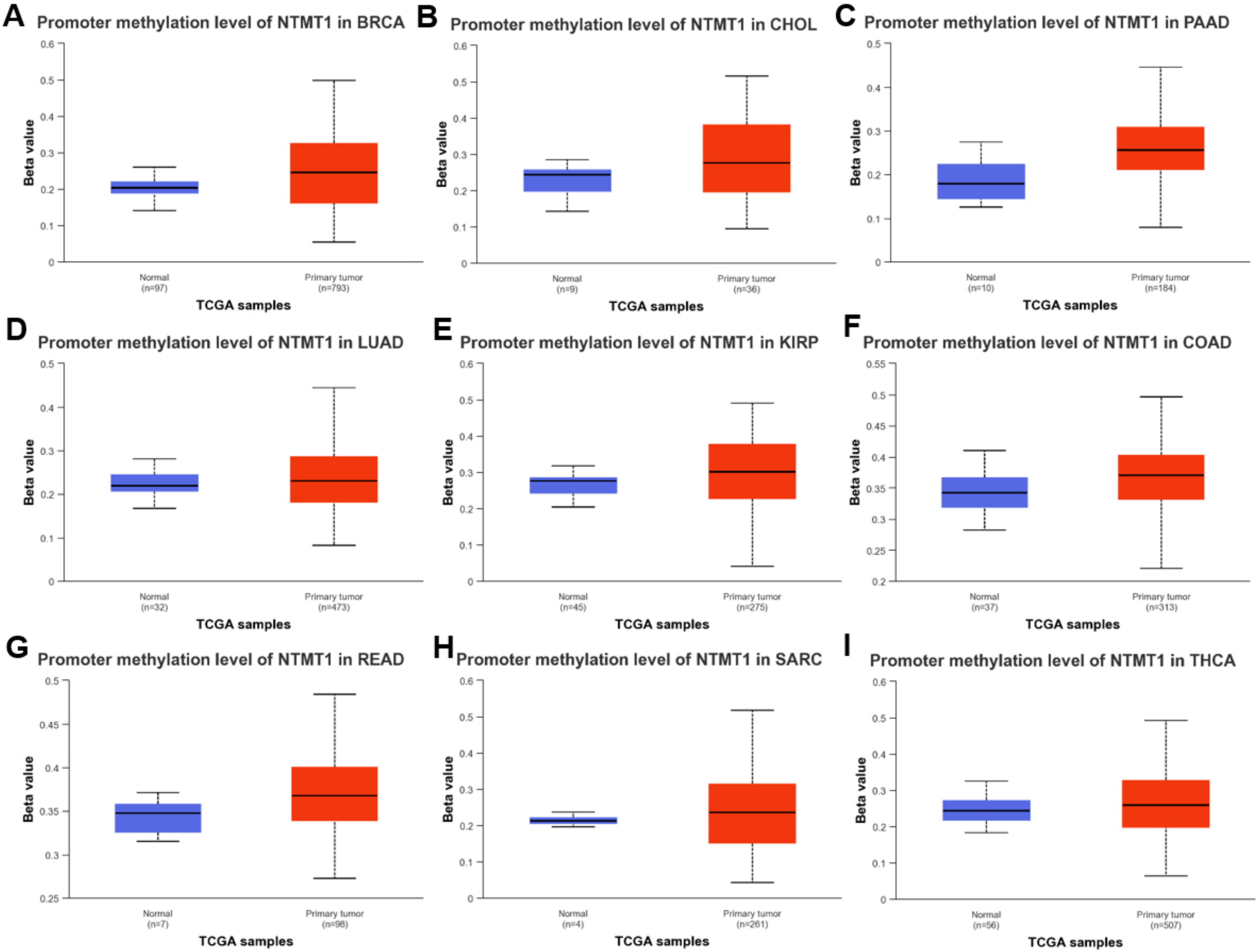
A
Promoter methylation level of NTMT1 in BRCA
Beta value
0
0.1
0.2
0.3
0.4
0.5
0.6
Normal
(n=97)
Primary tumor
(n=793)
TCGA samples
B
Promoter methylation level of NTMT1 in CHOL
Beta value
0
0.1
0.2
0.3
0.4
0.5
0.6
Normal
(n=9)
Primary tumor
(n=36)
TCGA samples
C
Promoter methylation level of NTMT1 in PAAD
Beta value
0
0.1
0.2
0.3
0.4
0.5
Normal
(n=10)
Primary tumor
(n=184)
TCGA samples
D
Promoter methylation level of NTMT1 in LUAD
Beta value
0
0.1
0.2
0.3
0.4
0.5
Normal
(n=32)
Primary tumor
(n=473)
TCGA samples
E
Promoter methylation level of NTMT1 in KIRP
Beta value
0
0.1
0.2
0.3
0.4
0.5
0.6
Normal
(n=45)
Primary tumor
(n=275)
TCGA samples
F
Promoter methylation level of NTMT1 in COAD
Beta value
0.2
0.25
0.3
0.35
0.4
0.45
0.5
0.55
Normal
(n=37)
Primary tumor
(n=313)
TCGA samples
G
Promoter methylation level of NTMT1 in READ
Beta value
0.25
0.3
0.35
0.4
0.45
0.5
Normal
(n=7)
Primary tumor
(n=98)
TCGA samples
H
Promoter methylation level of NTMT1 in SARC
Beta value
0
0.1
0.2
0.3
0.4
0.5
0.6
Normal
(n=4)
Primary tumor
(n=261)
TCGA samples
I
Promoter methylation level of NTMT1 in THCA
Beta value
0
0.1
0.2
0.3
0.4
0.5
0.6
Normal
(n=56)
Primary tumor
(n=507)
TCGA samples
