# Supplementary material for: The comprehensive analysis of the prognostic and functional role of N-terminal methyltransferases 1 in pan-cancer
Source: PeerJ. 2023 Oct 24;11:e16263. doi: 10.7717/peerj.16263 (PMC10607204; doi:10.7717/peerj.16263)
Supplement: Supplemental Information 5 [file peerj-11-16263-s005.pdf]

Gene Symt	Gene ID
SURF2	ENSG00000148291.9
PPM1G	ENSG00000115241.10
ZMYND19	ENSG00000165724.5
PSMB7	ENSG00000136930.12
RUVBL2	ENSG00000183207.12
ELOF1	ENSG00000130165.10
DRG1	ENSG00000185721.11
TUBG1	ENSG00000131462.7
SSNA1	ENSG00000176101.11
MRGBP	ENSG00000101189.6
CDKN3	ENSG00000100526.19
SWI5	ENSG00000175854.11
RPL26L1	ENSG00000037241.7
WDR5	ENSG00000196363.9
PDXK	ENSG00000160209.18
REXO4	ENSG00000148300.11
TBL2	ENSG00000106638.15
AURKAIP1	ENSG00000175756.13
NELFE	ENSG00000204356.11
ARPC5L	ENSG00000136950.13
TUBB4B	ENSG00000188229.5
WDR53	ENSG00000185798.7
RANGAP1	ENSG00000100401.19
LYAR	ENSG00000145220.13
BRAP	ENSG00000089234.15
LRWD1	ENSG00000161036.10
ZNHIT2	ENSG00000174276.6
PMPCA	ENSG00000165688.11
THOP1	ENSG00000172009.14
EFCAB11	ENSG00000140025.15
ODF2	ENSG00000136811.16
HMOX2	ENSG00000103415.11
GTF2A2	ENSG00000140307.10
SAC3D1	ENSG00000168061.13
WDR62	ENSG00000075702.16
FAM104A	ENSG00000133193.12
LMNB2	ENSG00000176619.10
MED27	ENSG00000160563.13
UBE2J2	ENSG00000160087.20
ELP5	ENSG00000170291.14
FAM220A	ENSG00000178397.12
C16orf95	ENSG00000260456.6
SLC39A3	ENSG00000141873.10
POLR2D	ENSG00000144231.10
SLC35E4	ENSG00000100036.12
LRR1	ENSG00000165501.16
NSUN4	ENSG00000117481.10
RP1-198K1	ENSG00000275457.1
CLPB	ENSG00000162129.12
NOC4L	ENSG00000184967.6
RNF114	ENSG00000124226.10
NOSIP	ENSG00000142546.13
AC005076.	ENSG00000224046.1

LACE1	ENSG00000135537.16
CREM	ENSG00000095794.19
MEA1	ENSG00000124733.3
PSMG1	ENSG00000183527.11
RP1-191J1	ENSG00000272476.1
AC002467.	ENSG00000241764.3
CCT7	ENSG00000135624.15
SLC38A7	ENSG00000103042.8
COPRS	ENSG00000172301.10
CETN3	ENSG00000153140.8
FAM92A1	ENSG00000188343.12
NDUFA8	ENSG00000119421.6
SLC2A8	ENSG00000136856.17
MLF1	ENSG00000178053.17
MAPKAP1	ENSG00000119487.16
SPATA33	ENSG00000167523.13
HSF2BP	ENSG00000160207.8
C9orf40	ENSG00000135045.6
NFKBIB	ENSG00000104825.16
GPR137	ENSG00000173264.13
POC1A	ENSG00000164087.7
PGP	ENSG00000184207.8
KDM4D	ENSG00000186280.6
KIF2C	ENSG00000142945.12
LRRC73	ENSG00000204052.4
DNAL1	ENSG00000119661.14
PSMC3IP	ENSG00000131470.14
RP11-961A	ENSG00000277597.1
CMC2	ENSG00000103121.8
MED26	ENSG00000105085.10
CEP83-AS1	ENSG00000278916.1
POMT2	ENSG00000009830.11
RP11-61N2	ENSG00000226268.3
RP11-96K1	ENSG00000273010.1
FAM229B	ENSG00000203778.7
TMPOP2	ENSG00000262904.1
LINC00467	ENSG00000153363.12
RP11-524C	ENSG00000260830.1
TEX30	ENSG00000151287.16
APITD1-CC	ENSG00000251503.7
DBF4P1	ENSG00000235489.4
THUMPD3	ENSG00000134077.15
TTLL5	ENSG00000119685.19
PKMYT1	ENSG00000127564.16
FBXW5	ENSG00000159069.13
PDRG1	ENSG00000088356.5
C11orf71	ENSG00000282682.1
